# Chromatin remodeling by the histone methyltransferase EZH2 drives lung pre-malignancy and is a target for cancer prevention

**DOI:** 10.1186/s13148-021-01034-4

**Published:** 2021-02-25

**Authors:** Carmen S. Tellez, Maria A. Picchi, Daniel Juri, Kieu Do, Dhimant H. Desai, Shantu G. Amin, Julie A. Hutt, Piotr T. Filipczak, Steven A. Belinsky

**Affiliations:** 1 Lung Cancer Program, Lovelace Respiratory Research Institute, 2425 Ridgecrest Drive SE, Albuquerque, NM 87108 USA; 2 Department of Pharmacology, Penn State College of Medicine, Hershey, PA USA

**Keywords:** EZH2, DZNep, Lung cancer

## Abstract

**Background:**

Trimethylation of lysine 27 and dimethylation of lysine 9 of histone-H3 catalyzed by the histone methyltransferases EZH2 and G9a impede gene transcription in cancer. Our human bronchial epithelial (HBEC) pre-malignancy model studied the role of these histone modifications in transformation. Tobacco carcinogen transformed HBEC lines were characterized for cytosine DNA methylation, transcriptome reprogramming, and the effect of inhibiting EZH2 and G9a on the transformed phenotype. The effects of targeting EZH2 and G9a on lung cancer prevention was assessed in the A/J mouse lung tumor model.

**Results:**

Carcinogen exposure induced transformation and DNA methylation of 12–96 genes in the four HBEC transformed (T) lines that was perpetuated in malignant tumors. In contrast, 506 unmethylated genes showed reduced expression in one or more HBECTs with many becoming methylated in tumors. ChIP-on-chip for HBEC2T identified 327 and 143 genes enriched for H3K27me3 and H3K9me2. Treatment of HBEC2T and HBEC13T with DZNep, a lysine methyltransferase inhibitor depleted EZH2, reversed transformation, and induced transcriptional reprogramming. The EZH2 small molecule inhibitor EPZ6438 also affected transformation and expression in HBEC2T, while a G9a inhibitor, UNC0642 was ineffective. Genetic knock down of EZH2 dramatically reduced carcinogen-induced transformation of HBEC2. Only DZNep treatment prevented progression of hyperplasia to adenomas in the NNK mouse lung tumor model through reducing EZH2 and affecting the expression of genes regulating cell growth and invasion.

**Conclusion:**

These studies demonstrate a critical role for EZH2 catalyzed histone modifications for premalignancy and its potential as a target for chemoprevention of lung carcinogenesis.

## Background

Lung cancer is responsible for more than 1.5 million deaths globally [1]. While smoking cessation reduces mortality, 50% of lung cancer cases are diagnosed in former smokers, necessitating the need for effective preventive agents [2]. Developing lung cancer preventive therapies has been challenging due to the heterogeneity of this disease with respect to genetic and epigenetic alterations and the need for drugs to be non-genotoxic.

Epigenetic deregulation involving the methylation of cytosine to form 5-methylcytosine in conjunction with histone modifications and nucleosome remodeling in gene promoters to silence transcription is a key step in initiation and pre-malignancy affecting genes and microRNAs (miRs) involved in all aspects of cell regulation [3–5]. With respect to lung cancer, two histone modifications implicated in the formation of a transcriptionally repressive chromatin state involve trimethylation at Lysine 27 and dimethylation at Lysine 9 of histone H3 (H3K27me3 and H3K9me2) via S-adenosyl-methionine-dependent methylation catalyzed by the histone methyltransferases (HMTs) EZH2 and G9a. Increased expression of EZH2 appears often in solid tumors including lung and during development of squamous cell carcinoma [6, 7]. SiRNA inhibition of EZH2 induced apoptosis of oncogenetically transformed human bronchial epithelial cells [8]. G9a expression is also increased significantly in lung cancer, its over expression promotes cell invasion and metastasis, and its silencing in transformed bronchial epithelial cells induces G1 arrest [8, 9].

Our group has identified key molecular changes driving transformation and clonal outgrowth of pre-neoplastic cells during exposure to carcinogens in tobacco smoke using an in vitro model of *hTERT/CDK4* immortalized human bronchial epithelial cells (HBECs). Transformation is epigenetically driven through increased DNMT1 protein, can be attenuated by overexpression of the de novo cytosine methyltransferase DNMT3b, and involves epithelial to mesenchymal transition (EMT [10–12]). While we have evaluated individual gene promoter hypermethylation by methylation specific PCR and genome-wide methylation using the Illumina HM450K BeadChiP array, a key question not addressed globally is the timing between reduced transcription and the acquisition of dense promoter hypermethylation. Our study of EMT showed that enrichment of H3K27me3 at the miR-200b, miR-200c, and miR-205 promoters was strongly associated with loss of expression and preceded dense promoter methylation that was not observed until malignancy [11]. If the epigenome in pre-malignancy is largely repressed by histone modifications rather than methylation, targeting EZH2 and/or G9a may offer a great opportunity to reverse gene expression to impede progression to malignancy.

The importance of EZH2 and G9a in the induction of heterochromatin has made them attractive targets for therapy. This has spurred the development of small molecule inhibitors targeting EZH2 and G9a and offers the opportunity to evaluate the effects of these drugs on reversing gene silencing and transformation in our pre-malignancy model. Epizyme 6438 (EPZ6438) is a selective inhibitor of wild type and mutant proteins and has shown activity in modulating the growth of SMARCB1 mutant malignant rhabdoid tumors and EZH2 mutant non-Hodgkin lymphoma [13, 14]. The G9a inhibitor UNC0642 is an inhibitor of this HMT and shows efficacy in breast cancer xenografts [15, 16]. 3-Deazaneplanocin A (DZNep) was developed as an epigenetic anticancer drug. DZNep inhibits S-adenosylhomocysteine hydrolase leading to the indirect inhibition of S-adenosyl-methionine-dependent methylation to reduce levels of H3K27me3 and also promotes proteasome-dependent degradation of EZH2 [17, 18]. DZNep induces cell cycle arrest and apoptosis of acute myelogenous leukemia cells and inhibits growth of non-small cell lung cancer cells [19, 20]. The omega-3 polyunsaturated fatty acids (*n*-3 PUFAs) eicosapentaenoic acid (EPA) and docosahexaenoic acid (DHA) induce degradation of EZH2 through a posttranslational mechanism via a proteasome-mediated pathway [21]. A fish oil diet rich in *n*-3 PUFAs reduced colonic polyps and adenomas in dimethylhydrazine-induced colon cancer in rats, was associated with lower risk of breast cancer, and a significant 63% reduction in prostate cancer-specific mortality [22–24]. Thus, modulation of HMTs for prevention can be potentially achieved with drug, small molecule, or dietary intervention.

The A/J mouse is a sensitive strain for lung cancer induction by carcinogens such as NNK, making it ideal for testing the efficacy of drugs or dietary supplements in modulating tumor development [25, 26]. We have characterized the timing for tumor development in the mouse lung that begins with alveolar type II hyperplasia and progresses to adenoma and ultimately carcinoma [25]. Our initial studies demonstrated that treatment with NNK induces mutations in codon 12 of the K-ras oncogene [27]. Subsequent studies demonstrated a major contribution of DNMT1 and gene methylation to tumor formation by showing that knocking out one copy of this gene reduced the incidence of NNK-induced lung tumors by 50%, and treatment with a demethylating agent and histone deacetylase inhibitor significantly reduced tumor formation [28, 29].

The goals of this study were to first expand our in vitro transformation model to include additional HBEC lines and then to characterize the relationship between genome-wide effects on transcription and methylation. Second, the effect of inhibiting EZH2 and G9a on in vitro transformation and in vivo tumor development was evaluated using EPZ6438 and UNC0642, DZNep, and omega-3 fatty acids.

## Results

### Transformation of immortalized bronchial epithelial cell lines

**Fig. 1:**
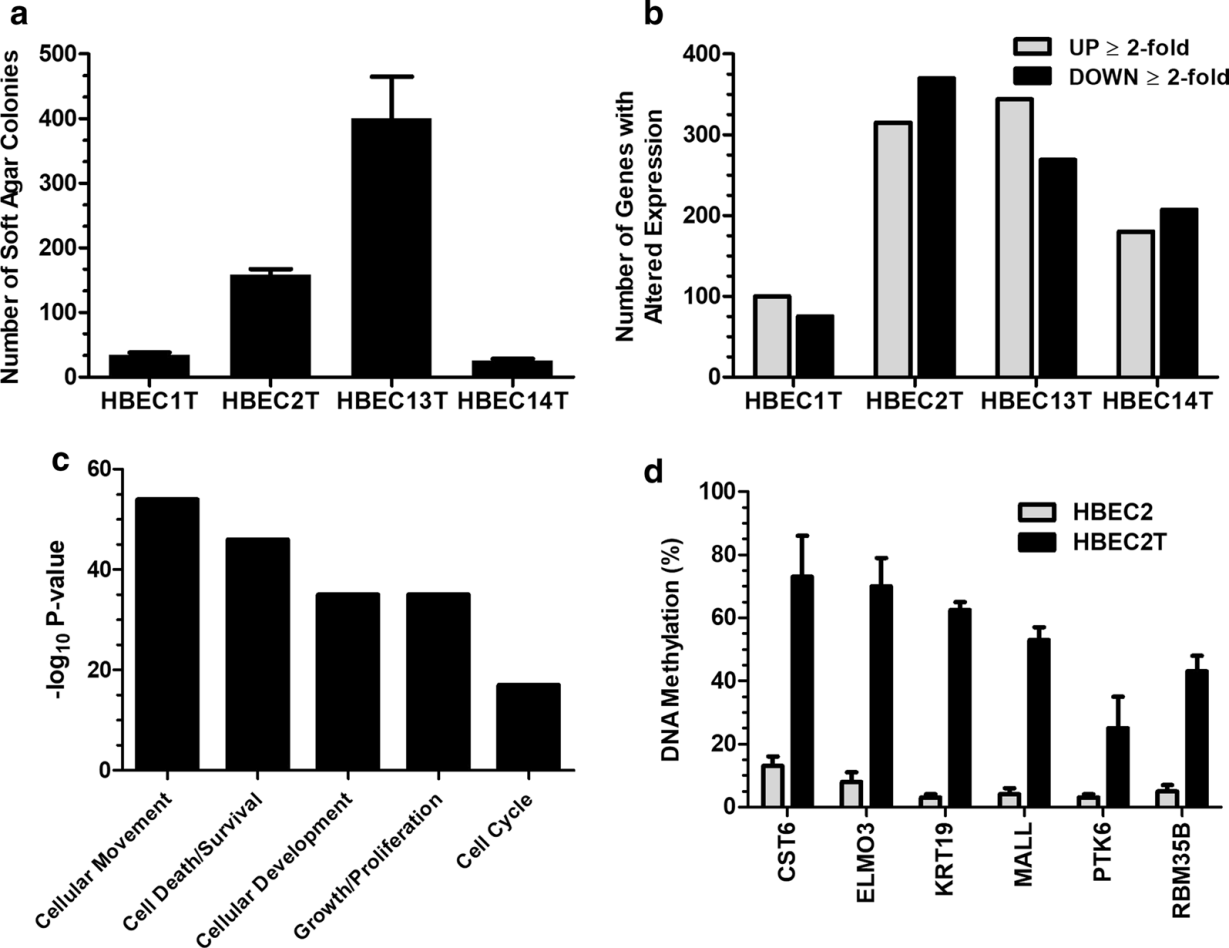
Carcinogen-induced transformation and epigenetic reprogramming of HBEC lines. Comparison of colony formation across HBEC lines after carcinogen treatment (**a**). The number of genes with increased or decreased expression across transformed HBECs (**b**). Ingenuity Pathway Analysis was used to identify pathways for the 1369 differentially expressed genes in one or more transformed HBECs (**c**). Bisulfite sequencing of promoter regions of CST6, ELMO3, KRT19, MALL, PTK6, and RBM35B in HBEC2 and HBEC2T shows increased DNA methylation resulting from transformation (**d**). Mean ± SEM of ten clones

Prior studies examining the effect of carcinogen treatment on transformation of HBEC1 and HBEC2 were extended to HBEC13 and HBEC14 [10, 11]. Following 12 weeks of carcinogen treatment, colony formation that is indicative of cell transformation was apparent in both cell lines with no colonies formed from the vehicle controls (Fig. 1a). The 25 colonies formed from HBEC14 cells approximated that seen for HBEC1, while 400 colonies were seen with HBEC13, a 2.5-fold greater transformation efficiency than seen for HBEC2 (Fig. 1a).

### Global reprogramming of the transcriptome and induction of gene promoter hypermethylation in transformed clones

The effect of transformation on global gene expression (≥ 2-fold change) was assessed in HBEC lines using Illumina Whole-Genome Expression BeadChip. The number of differentially expressed genes generally correlated with transformation efficiency with an equal number of genes showing increased or decreased expression (Fig. 1b). Transformed HBEC1 and HBEC14 (designated HBEC1T and HBEC14T) had 175 and 387 genes with altered expression, while ~ 650 genes were differentially expressed in HBEC2T and HBEC13T. Pathway analysis for the 1369 genes differentially expressed in one or more transformed cell line showed highly significant alterations in molecular and cellular functions involved in cellular movement, cell death/survival; cell development, growth/proliferation; and cell cycle (Fig. 1c).

**Fig. 2:**
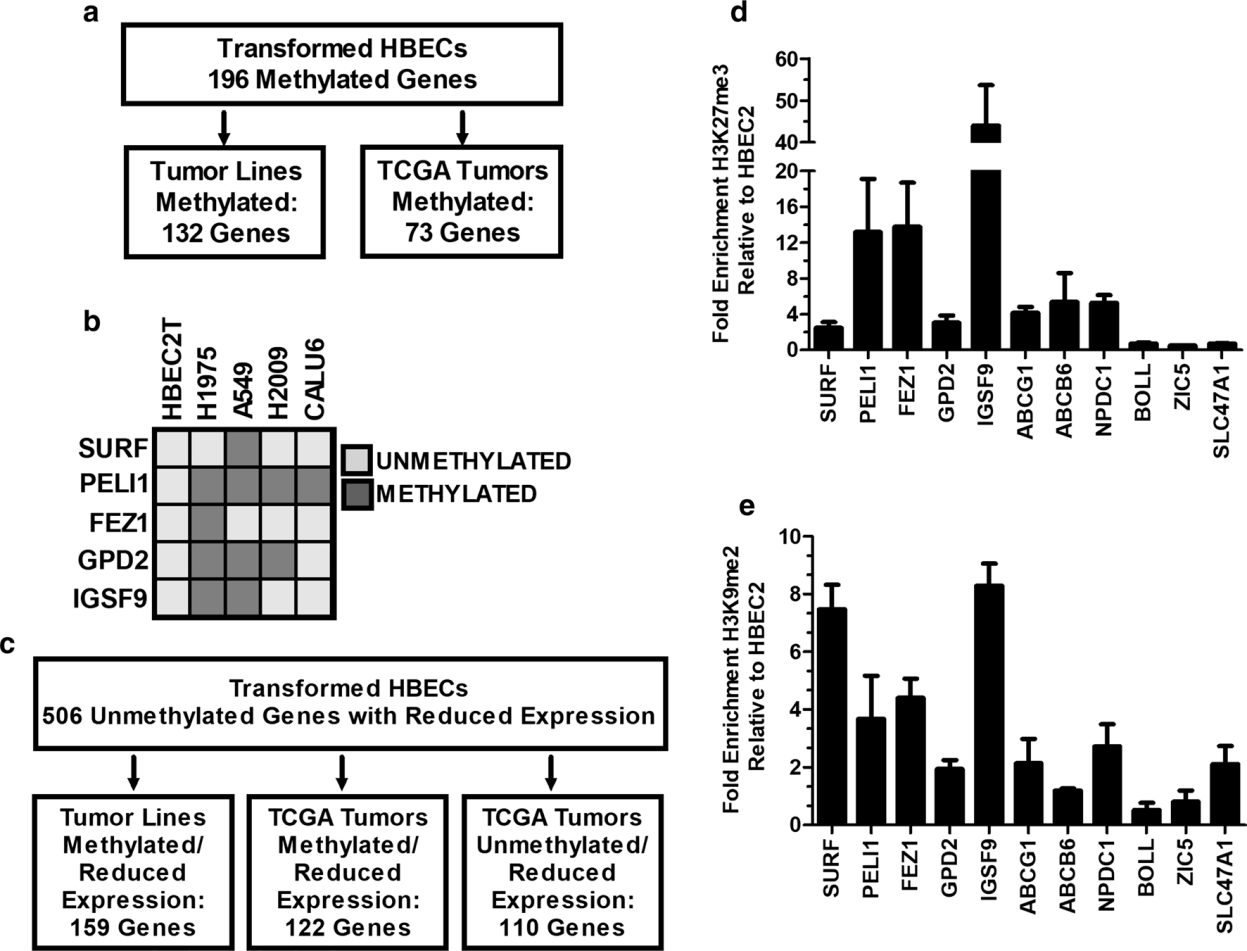
Perpetuation and progression of epigenetic changes in tumors and chromatin remodeling in HBECT. Flow diagram depicting the number of genes methylated in transformed HBEC lines that are also methylated in tumor lines and TCGA tumors (**a**). Combined bisulfite restriction analysis comparing methylation of SURF, PELI1, FEZ1, GPD2, and IGSF9 between HBEC2T and the tumor lines H1975, A549, H2009, and Calu6 (**b**). Flow diagram depicting the number of unmethylated genes with reduced expression in transformed HBECs that become methylated in tumor lines and TCGA tumors or retain reduced expression in tumors (**c**). Chromatin immunoprecipitation showing enrichment of H3K27me3 (**d**) or H3K9me2 (**e**) in HBEC2T relative to HBEC2 for 11 genes listed on the *x*-axis. Values are mean ± SEM for ChIP-qPCR conducted in triplicate

The pre-malignancy model developed through exposure of HBEC lines to DNA damaging carcinogens is epigenetically driven based on the requirement for DNMT1 and acceleration of transformation by overexpression of the de novo methyltransferase DNMT3b that together can lead to methylation of cytosines in promoter regions and gene silencing [10, 12]. DNMT1 also participates in transcriptional repression by its presence in chromatin remodeling complexes with the histone methyltransferases EZH2 and G9a whose methylation at histone H3 lysine 27 and lysine 9 reduce gene transcription [30–32]. Thus, we first evaluated whether the large number of genes with reduced expression stemmed from hypermethylation within promoter regions. Surprisingly, there was a low frequency of gene methylation detected that affected 12, 49, 65, and 96 genes in HBEC1, HBEC14, HBEC13, and HBEC2 transformed cell lines, respectively. Six genes with hypermethylation in HBEC2T based on the HM450K array were randomly selected and bisulfite sequencing validated dense methylation of CpGs within their promoter regions (Fig. 1d, Additional file 1: Fig. S1). The methylation state of the 196 genes methylated in one or more of the transformed HBEC lines was also assessed in 19 lung tumor derived cell lines and in 811 tumors from The Cancer Genome Atlas (TCGA). Among these 196 genes, 132 and 73 were methylated in ≥ 15% of the tumor lines and tumors from TCGA (Fig. 2a, Additional file 1: Table S1).

### Reduced gene expression and associated chromatin remodeling predispose to gene methylation

Our previous studies on epigenetic silencing of microRNAs in the HBEC transformation model showed that chromatin mediated transcriptional repression preceded the acquisition of dense promoter hypermethylation [11, 33]. Thus, five genes with reduced expression and not methylated in HBEC2T based on HM450K array data were selected for evaluation of methylation in H1975, A549, H2009, and Calu6 tumor lines by Combined Bisulfite Restriction Analysis (COBRA). All genes were confirmed as unmethylated in HBEC2T, but were methylated in one or more of the four cell lines evaluated (Fig. 2b). Therefore, this approach was extended to the 506 genes not methylated, but with reduced expression in one or more transformed HBEC line. These genes were assessed for methylation status using prior HM450K data from lung tumor cell lines and RNA-seq data from tumor-normal pairs (*n* = 102) from TCGA. There were 159 (31%) and 122 (24%) genes with reduced expression that were methylated in ≥ 15% of tumor lines and primary tumors (Fig. 2c; Additional file 1: Table S2). Finally, for the 384 unmethylated genes with reduced expression in HBECTs, but not methylated in tumors, 110 were found to have reduced expression ≥ 1.5-fold in TCGA (FDR < 0.05 [Fig. 2c**]**).

HBEC2T cells were subjected to ChIP-on-chip to globally identify genes enriched for H3K27me3 and H3K9me2. There were 327 genes with ≥ 2-fold enrichment for H3K27me3, of which 67% also showed ≥ 2-fold reduction in expression (Additional file 1: Table S3). In contrast 143 genes were enriched for H3K9me2 with only 4 genes showing reduced expression (Additional file 1: Table S4).

ChIP was performed to validate enrichment for H3K27me3 and/or H3K9me2. Five genes selected were shown to be unmethylated in HBEC2T, but methylated in tumor lines (Fig. 2b). H3K27me3 and H3K9me2 were enriched 3.5–55-fold and 2–8-fold in the gene promoters with enrichment for H3K27me3 greater than H3K9me2 for all genes except SURF (Fig. 2d, e). ABCG1, ABCG6, and NPDC1 were enriched for both marks based on ChIP-on-chip data and this was confirmed by ChIP (Fig. 2d, e). BOLL, ZIC, and SLC47A were only enriched for H3K9me2 and this was also validated (Fig. 2d, e).

### Inhibiting EZH2 reduces HBEC transformation through reprogramming the transcriptome

**Fig. 3:**
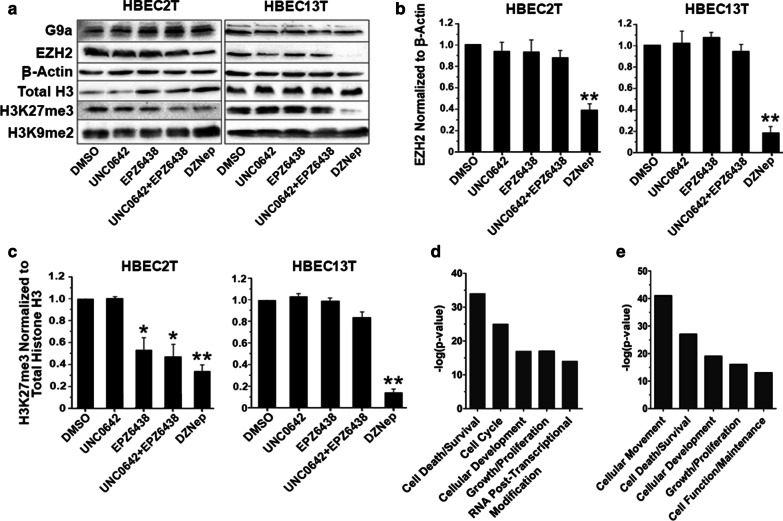
DZNep treatment of transformed HBEC lines reprograms the transcriptome through chromatin remodeling affecting EZH2 and H3K27me3. Western blot showing the effect of treatment of HBEC2T and HBEC13T for 8 days with DMSO, UNC0642, EPZ6438, UNC0642 + EPZ6438, or DZNep on levels of G9a, EZH2, β-Actin, total histone H3, H3K27me3, and H3K9me2 (**a**). Densitometry quantitation of EZH2 (**b**) and (**c**) H3K27me3 levels in HBEC2T and HBEC13T cells treated with chromatin remodeling agents. Values are mean ± SEM (*n* = 2), *p < 0.05; **p < 0.01 compared to β-Actin or total histone H3. Ingenuity Pathway Analysis was used to identify pathways for differentially expressed genes following treatment of HBEC2T and HBEC13T with DZNep (**d**) or EPZ6438 + UNC0642 (**e**)

Transcriptome and ChIP-on-chip studies support chromatin remodeling as a likely driver for transformation in the HBEC pre-malignancy model. Thus, the effect of targeting EZH2 and/or G9a on reversing transformation was evaluated in HBEC2T and HBEC13T cell lines that showed the highest transformation frequency and the largest number of genes with differential expression. Dose range finding studies were conducted for the EZH2 inhibitor EPZ6438, the G9a inhibitor UNC0642, the S-adenosylhomocysteine hydrolase inhibitor DZNep, and the omega-3-acid ethyl esters drug Lovaza to identify non-cytotoxic doses with minimal effects on cell growth (< 10% reduction). HBECT lines were treated for 8-days to allow for evaluating treatment effects on H3K27me3 turnover that can take approximately 1 week [34]. DZNep (0.25 µM) proved to be the most potent inhibitor, reducing transformation by 80% for both HBECT lines (Table 1). Inhibiting G9a with UNC0642 (2 µM) had no effect on transformation, while EPZ6438 (2 µM) reduced transformation 70% for HBEC2T, but had no effect on HBEC13T. Combining EPZ6438 with UNC0642 reduced transformation 70% and 48% for HBEC2T and HBEC13T (Table 1). Treatment with Lovaza had a modest (30%) effect on reducing transformation. Western blot analysis showed that treatment with DZNep reduced levels of EZH2 in HBEC2T and HBEC13T by 60% and 80% and H3K27me3 by ≥ 75% (Fig. 3a–c). DZNep treatment had no effect on G9a and H3K9me2 (Fig. 3a, Additional file 1: Fig. S2). EPZ6438 alone or combined with UNC0642 had no significant effect on EZH2, but reduced H3K27me3 by 45% and 55% in HBEC2T (Fig. 3c). In contrast, EPZ6438 showed minimal potency toward affecting levels of H3K27me3 in HBEC13T with no effect or a 20% reduction in HBEC13T treated cells (Fig. 3c). UNC0642 did not cause any reduction in G9a or H3K9me2 (Fig. 3a). Lovaza also had no effect on either HMT or their regulated histones (Additional file 1: Fig. S3).

**Table 1:** Histone methyltransferase inhibitors reduce transformation of HBECs in conjunction with reprogramming of the transcription

Cell line	Treatment	Transformation^a^	50–100%	> 100%	Total
		(% Reduction)		(Change in expression # genes)	
HBEC2T	DZNep	80 ± 2	1675	976	2651
	EPZ6438	70 ± 3	328	160	488
	UNC0642	0	85	23	108
	EPZ/UNC	70 ± 4	574	323	897
	Lovaza	30 ± 3	20	2	22
HBEC13T	DZNep	80 ± 3	838	332	1170
	EPZ6438	0	211	76	287
	UNC0642	0	80	25	153
	EPZ/UNC	48 ± 4	265	109	374
	Lovaza	28 ± 3	22	1	23

^a^Mean ± SD % reduction in transformation from triplicate soft agar assays compared to vehicle

The effect of these agents on the transcriptome was evaluated through comparing vehicle to the 8-day treated cell lines. The number of genes with 50–100% and > 100% change in expression of either direction was enumerated across treatment groups. Consistent with potency for inhibiting transformation, DZNep treatment was associated with the greatest number of differentially expressed genes (2921 for HBEC2T; 1170 for HBEC13T [Table 1]). Combined therapy with EPZ6438/UNC0642 in HBEC2T also led to a change in expression for 897 genes, while only a modest number of genes were affected when these inhibitors were used as single agents (Table 1). Lovaza had almost no effect on gene expression. Pathway analysis for genes differentially expressed in HBEC2T and HBEC13T following DZNep treatment showed highly significant effects in molecular and cellular functions (top five described) involved in cell death/survival; cell cycle, cell development, growth/proliferation; and RNA post-transcriptional modifications (Fig. 3d). Three of the same cellular functions were also altered following combined treatment with EPZ6438/UNC0642 (cell death/survival, development, growth/proliferation) while cell movement and function also comprised the top five processes affected by this treatment (Fig. 3e).

**Fig. 4:**
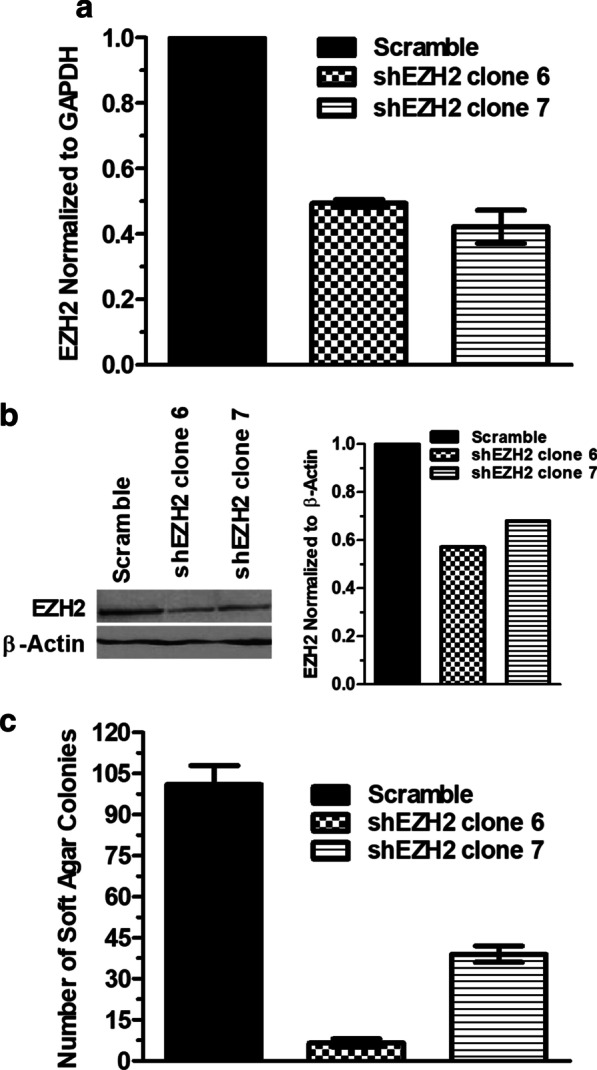
Genetic knock down of EZH2 attenuates carcinogen-induced transformation. shEZH2 knock out clones 6 and 7 show reduced transcription determined by RT-qPCR (**a**) and reduced levels of protein by Western blot compared to scrambled control (**b**). Protein levels of EZH2 were quantified by densitometry relative β-Actin (**b**). Comparison of colony formation between scrambled control and EZH2 knock down clones following 12 weeks of carcinogen treatment (**c**)

The effectiveness of DZNep and EPZ6438 on reversing transformation and gene re-expression support a major role for EZH2 in pre-malignancy. To validate this premise, the effect of genetic knock down of EZH2 was studied in HBEC2 that was sensitive to inhibition by either agent. Because complete knock out of EZH2 is associated with inhibition of proliferation and induces senescence, the two stable shEZH2 clones selected showed reduced transcription of 51% and 58% and a 43% or 32% reduction in levels of protein with no effect on cell growth (Fig. 4a, b [34, 35]). The HBEC2 scrambled control and shEZH2 clones 6 and 7 were treated with vehicle or MNU/BPDE once a week for 12 weeks and evaluated for transformation via growth in soft agar. No colonies were seen in vehicle control cells, while knock down of EZH2 largely prevented (clone 6) or reduced transformation by 61% for shEZH2 clone 7 compared to the scrambled control (Fig. 4c).

### DZNep treatment reduces lung cancer progression in the A/J mouse model

In vitro studies suggest that modulating EZH2 could be used to prevent progression of preneoplastic lung lesions. This hypothesis was tested in the A/J mouse model, where treatment with NNK leads to the development of alveolar type II cell hyperplasia that progresses to adenoma and ultimately adenocarcinoma [25, 26]. The ability of DZNep (2 mg/kg, 5 days/week), combined treatment with EPZ6438/UNC0642 (75/5 mg/kg M, W, F), or Lovaza diet (4%) to prevent progression of hyperplasia to adenoma was evaluated by starting treatment 20 weeks post exposure to NNK, a period of time during which predominantly hyperplasia have developed within the lung parenchyma [24, 25]. Doses selected and treatment schedule were based on prior studies that did not report any significant toxicities from the dosing regimen [14, 16, 36–38]. In addition, a pilot study exposing mice for 2 weeks to the selected dosing regimen for DZNep and EPZ6438/UNC0642 was conducted with no effect on weight or activity. Lhuissier et al. [38] also showed no effect on cognitive function, histopathology assessed for lung, kidney, brain, liver, and heart, or hematology in mice treated for 8 weeks with 2 mg/kg DZNep, supporting selection of this dose for prevention studies. Mice were treated for 20 weeks followed by sacrifice and enumeration of tumor multiplicity. Histological assessment revealed that DZNep treatment reduced the progression of hyperplasia to adenoma by 55%, while the EPZ6438/UNC0642 combination or Lovaza diet did not affect progression (Table 2). There was no effect on morphology of the adenomas that did occur in the DZNep-treated group, nor did their size differ from the vehicle group. None of the other treatments affected morphology or size of the adenomas. Furthermore, only a modest affect on weight gain of 8% was observed between mice treated with vehicle versus DZNep or EPZ6438/UNC0642. The Lovaza diet had no effect on weight gain compared to the control diet. In addition, none of the mice across treatment groups showed any change in activity or coat quality. There was also no evidence of toxicity to histologically appearing normal lung tissue from treated mice.

**Table 2:** DZNep Treatment Blocks Tumor Progression in the NNK A/J Mouse Model

Treatment	Hyperplasia	Adenoma	Carcinoma	Total lesions
Vehicle	4.3 ± 2.6	5.6 ± 2.4	0.3 ± 0.5	10.2 ± 2.8
DZNep	8.1 ± 3.0*	2.5 ± 2.0*	0.0 ± 0	10.6 ± 3.6
EPZ/UNC	5.2 ± 1.7	6.0 ± 2.9	2.1 ± 1.2	13.2 ± 3.8
Control Diet	4.5 ± 1.4	5.9 ± 3.4	0.9 ± 0.9	11.3 ± 3.8
Lovaza	6.6 ± 3.4	5.4 ± 1.8	1.4 ± 1.2	13.3 ± 4.1

Values are number of lesions (mean ± SD) from 12 to 15 mice per group

*p < 0.01 compared to vehicle

**Fig. 5:**
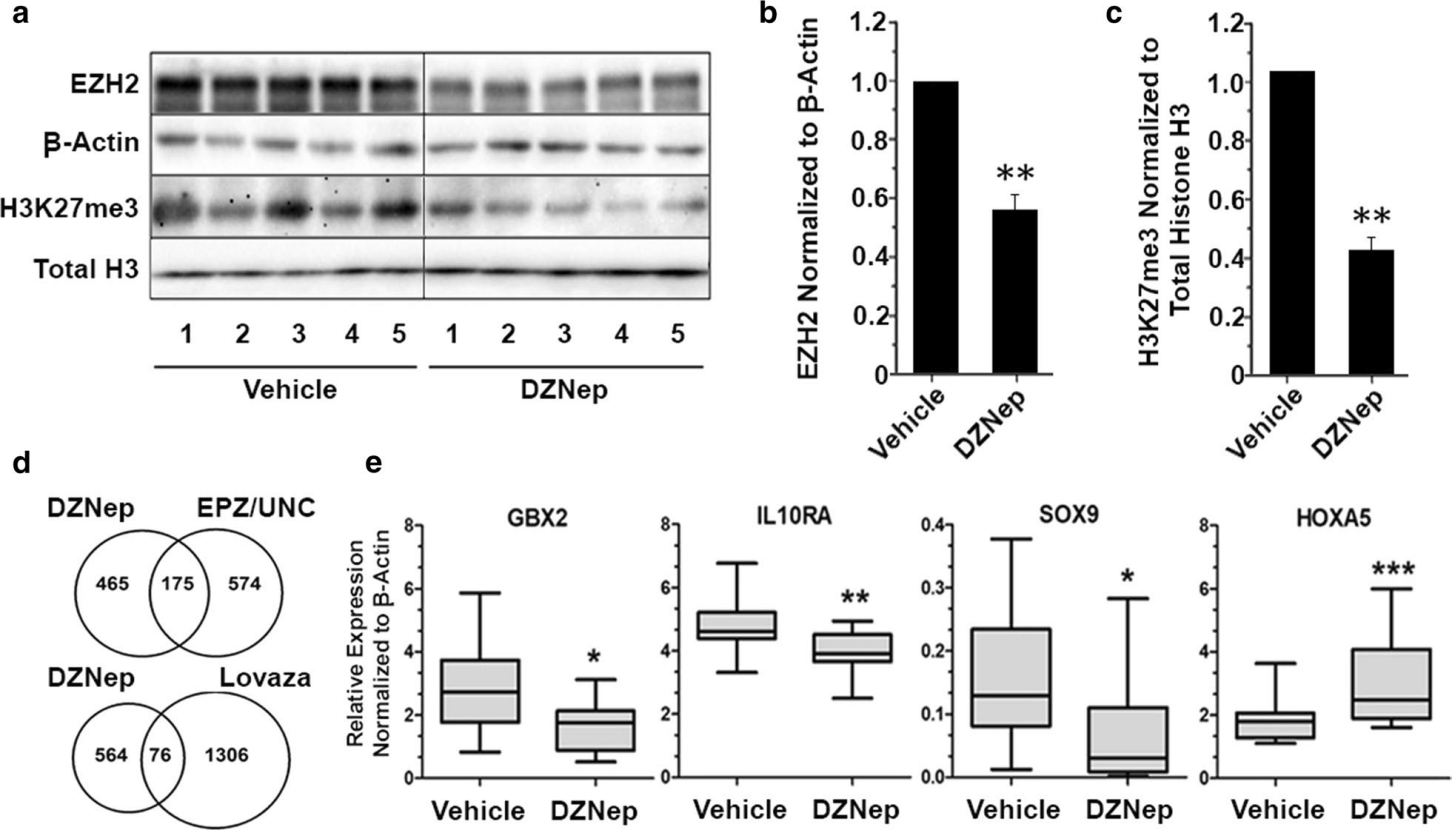
Chronic DZNep treatment reduces EZH2 and H3K27me3 levels while reprogramming the transcriptome in tumors induced by NNK in the A/J mouse lung. Western blot showing the levels of EZH2, β-actin, H3K27me3 and total histone H3 in tumors from vehicle and DZNep treated mice (**a**). Densitometry quantitation of EZH2 and H3K27me3 levels normalized to β-Actin and total histone H3 (**b**, **c**). Values are mean ± SEM from *n* = 10/group. *p < 0.05 compared to normal. Venn diagrams showing common genes (middle) with differential expression in tumors comparing DZNep to EPZ6438 + UNC0642 or Lovaza treatment (**d**). RT-qPCR for expression of GBX2, IL10RA, SOX9, and HOXA5 in tumors from vehicle and DZNep treated mice (**e**). Horizontal line within the boxes reflects the median and the whisker indicate the range (min to max) for relative levels of expression normalized to β-Actin. *p < 0.05, **p < 0.01, ***p < 0.001 compared to vehicle (*n* = 3)

Western blot comparing vehicle and DZNep tumors (*n* = 10/group) showed a 42% and 58% reduction in EZH2 and H3K27me3 (Fig. 5a–c, Additional file 1: Fig. S4). RNA-sequencing of vehicle and treatment tumors (*n* = 6/group) showed differential expression (increased or decreased with FDR ≤ 0.1) for 640 genes in DZNep treated tumors. A similar number of genes (749) were affected in tumors from EPZ6438/UNC0642 treated mice, while 1382 showed altered expression associated with the Lovaza diet. Venn diagrams were used to compare the overlap for gene expression changes between DZNep, that reduced tumor progression and modulated EZH2/H3K27me3 levels, to the other ineffective treatment groups. There were only 175 genes in common between DZNep and EPZ6438/UNC0642 treatment (Fig. 5d). Distinct expression profiles were seen between DZNep and Lovaza with only 76 genes commonly altered between the two treatments.

Given the effect of DZNep on EZH2, we determined which genes with altered expression were regulated by the polycomb repressive complex 2 (PRC2) in which EZH2 resides [37, 39]. There were 50 PRC2 target genes with altered expression ranging in magnitude from − 23 to 22 log2 fold change (Additional file 1: Table S5). Quantitative RT-PCR was performed for Gbx2, IL10ra, SOX9, and HOXA5 in vehicle and DZNep tumors (*n* = 10/group) to validate the expression changes detected by RNA-seq. These genes were selected because of their roles in regulating cell growth and invasion in lung and other cancers [40–44]. Consistent with RNA-seq results, RT-qPCR showed that DZNep treatment was associated with a significant reduction in expression for Gbx2, IL10ra, and SOX9 and a significant increase in expression for HOXA5 (Fig. 5e).

## Discussion

These studies show that tobacco carcinogen-induced transformation of HBEC lines is driven in part by chromatin remodeling mediated by EZH2 and its chromatin mark H3K27me3 that results in expression changes in genes involved in molecular and cellular functions associated with pre-malignancy. Epigenetic mediated gene silencing was also associated with promoter hypermethylation of ~ 200 genes across four HBECT lines, of which 40% and 70% were methylated in primary lung tumors and derived cell lines. Chromatin remodeling likely predisposes some genes to acquiring promoter hypermethylation as evident by ~ 30% of unmethylated genes in HBECT cells with reduced expression being methylated in tumors or cell lines. The translational impact of these findings has provided a new avenue for chemoprevention targeting EZH2 that is strongly supported by genetic knock down of this gene abrogating carcinogen-induced transformation and the ability of DZNep, through reducing levels of this HMT, to potently reverse HBEC transformation, prevent progression of pre-malignancy in the NNK A/J lung tumor model, and induce reprogramming of the transcriptome.

The prominent role for chromatin remodeling in initiating gene silencing and HBEC transformation is supported by basic and population-based studies. We provided the first potential link between double-strand break DNA damage and methylation through studying adenocarcinomas from plutonium-exposed workers and controls [45]. The frequency for p16 methylation in adenocarcinomas from the exposed workers increased as a function of plutonium lung dose. Subsequent studies demonstrated a highly significant association between double-strand break repair capacity measured in lymphocytes and the propensity for gene methylation detected in sputum from cancer-free smokers from the Lovelace Smokers Cohort [46]. Our initial studies demonstrating carcinogen-induced transformation of HBEC1 and HBEC2 also showed that transformation efficiency was inversely correlated with the formation of micronuclei resulting from double-strand break DNA damage [10]. Tobacco carcinogens induce double-strand breaks and DNMT1 as the major maintenance cytosine-methyltransferase maintains the epigenetic code during replication and following repair of DNA damage [47]. The repressive histone modifications H3K27me3 and H3K9me2 are also increased adjacent to double-strand breaks to affect chromatin conformation to impede transcription within the damaged region to allow for repair [48]. When a double-strand break is repaired, the epigenetic code is lost as there is no longer a hemi-methylated daughter strand for DNMT1 to copy. This can lead to “seeds of methylation” and persistence of repressive histone modifications [49]. O’Hagan et al. [50] corroborated this epigenetic response to DNA damage by inducing a double-strand break in an exogenous promoter of E-cadherin and showing recruitment of EZH2, DNMT1, and DNMT3B, the appearance of silencing histone modifications, and increasing DNA methylation in silent clones. However, many genes with reduced expression in our HBEC transformation model did not become methylated in primary tumors, but showed reduced expression. Kondo et al*.* [34] also found gene silencing in breast, prostate, and colon cancer by H3K27me3 can occur independent of promoter methylation.

Lung cancer takes 30 years or more to develop with malignancy arising from the extensive field cancerization that harbors epigenetic and genetic changes [51]. While targeting HMTs alone or in combination with DNA demethylating agents or histone deacetylase inhibitors has become a strategy in clinical trials for treatment of a variety of cancers, similar efforts for prevention have not been undertaken. Our studies provide the first proof-of-concept that effective targeting of EZH2 can reverse HBEC transformation and impede tumor development. DZNep at nanomolar concentrations proved to be the most potent agent through depleting EZH2 and inhibiting associated H3K27 trimethylation in HBEC2T and HBEC13T cells. Pathways most strongly affected were cell death and cell cycle, consistent with prior studies where DZNep inhibited growth of non-small cell lung cancer cells via induction of apoptosis and G1 cell cycle arrest [20]. EPZ6348 reduced H3K27me3 and was effective in reversing transformation of HBEC2T while HBEC13T was more resistant to this inhibitor. Differences in potency of EPZ6348 have been reported in studies of chronic lymphocytic leukemia cells where higher doses and extended treatment period were required to reduce levels of H3K27me3 and to affect survival [52]. The HBECT cells were resistant to UNC0642 as evident by a lack of its effect on transformation, G9a protein, and gene expression.

DZNep was the only effective intervention to block lung cancer development in the A/J mouse and the genes differentially expressed in treated pre-malignant lesions were largely distinct from the other interventions. DZNep through inhibiting S-adenosylhomocysteine hydrolase and inducing proteasome degradation reduces H3K27me3 and EZH2, respectively while EPZ6438 is a small molecule inhibitor of EZH2 that affects its catalytic activity to reduce H3K27me3 without effect on the levels of this HMT [13, 14, 17, 18]. This may account for the efficacy of DZNep for inhibiting adenoma formation and the marked difference in dose between DZNep (0.25 µM) and EZ6438 (2 µM) required for reversing transformation of HBECs in our in vitro studies. To demonstrate efficacy by EPZ6438 may require increasing the every-other day 75 mg/kg dose to a daily 250–350 mg/kg dosing regimen used to affect growth of rhabdoid and non-Hodgkin lymphoma tumors [13, 14]. This high dose is cost prohibitive and would also likely cause systemic toxicities prohibitive for a chronic prevention trial in smokers.

DZNep effectively reduced EZH2 to affect H3K27me3 levels, and modulated the expression of 50 PRC2 regulated genes. Expression changes detected by RNA-seq were validated in an expanded set of samples by RT-qPCR for IL10, SOX9, GBX2, and HOXA5, genes with biological functions that may contribute to the reduced progression of hyperplasia to adenoma. IL10 levels are increased in K-ras mutant mouse tumors and gene knock down inhibited tumor development while decreasing levels of infiltrating macrophages and T_reg_ lymphocytes [40]. Elevated expression of SOX9 drives EMT in lung cancer through Wnt/β-catenin pathway to promote migration and invasion [41]. Gastrulation brain homeobox 2 (GBX2) downregulation also suppresses proliferation, invasion, and angiogenesis of breast cancer cells through inhibiting Wnt/β-catenin signaling and reduces growth of prostate cancer xenografts [42, 43]. Finally, ectopic over expression of HOXA5, a master regulator of morphogenesis and cell differentiation that is commonly downregulated in lung cancers, inhibited cell proliferation and invasion [44]. Thus, the fact that treatment with DZNep also modulated these genes in directions associated with their inhibitory function on tumor development supports their involvement in blocking progression and provides some mechanistic insight into the effectiveness of this intervention. Together, these studies identify a potent inhibitory effect of DZNep through targeting EZH2 that could spur new strategies for developing an effective preventive intervention for lung carcinogenesis.

## Methods

Complete methods are provided in the online supplementary material.

### Cell culture, tumor specimens, and carcinogen exposure

HBEC1, HBEC2, HBEC13, and HBEC14 immortalized with *hTERT* and *CDK4* were obtained from Drs. Shay and Minna, Southwestern Medical Center, Dallas, TX. Cell culture conditions have been described [10]. Nineteen tumor-derived cell lines were obtained from the American Type Culture Collection.

Benzo(a)pyrene-diolepoxide1 (BPDE) and methylnitrosourea (MNU) were obtained from Drs. Desai and Amin (Penn State). HBEC13 and HBEC14 were exposed to 0.05 µM BPDE and 0.5 mM MNU for 1 h, once a week for 12 weeks, and propagated in keratinocyte specific medium (KSM) containing 15% FBS as described [10]. HBEC13 and HBEC14 were also treated with vehicle (saline) each week.

### Soft agar colony formation

Soft agar assays were conducted for carcinogen- and vehicle-treated cell lines as described previously [10]. Cultures were photographed and the colonies with diameters larger than 100 µm were counted using Image software.

### Animal treatment and histopathology

All animal procedures were conducted under protocols approved by the Institutional Animal Care and Use Committee and in concordance with National Institutes of Health guide for the care and use of Laboratory animals. Female A/J mice (*n* = 100) obtained from Jackson Laboratory (4–6 weeks old) were treated 3 times (every other day, 50 mg/kg, i.p.) with NNK dissolved in saline or with saline alone (0.1 ml). Animals were held for 20 weeks following carcinogen exposure to allow for the development of pre-invasive lesions: alveolar hyperplasia and adenoma [26]. Mice were then separated into 5 groups of 20 mice and treated for 20 weeks with individual or a combination of the agents. The agents used were DZNep (2.0 mg/kg, i.p.), EPZ6438 (75 mg/kg, i.p.), UNC0642 (5.0 mg/kg, i.p.), a diet rich with 4% omega-3 fish oil (EPA/DHA ethyl esters 45/37 provided by SOLUTEX GC, S.L.), or a control diet calorically balanced with 5% corn oil and 500 IU/g vitamin E (Additional file 1: Table S6). Mice were treated three times each week (Monday, Wednesday, Friday) with the combination of EPZ6438/UNC0642 to avoid toxicity by UNC0642 or five times each week with DZNep or vehicle (DMSO). Mice were fed control or 4% omega 3 fish oil diet continuously with fresh diet provided every 2 days.

Mice (12–15/group) were sacrificed by exsanguination and a single standardized section was prepared from all lungs that included the five lung lobes. Pulmonary lesions were classified as hyperplasia or neoplasia (adenoma or carcinoma) as described [25, 26]. Following sacrifice of the remaining 5 mice/group, tumors were removed from lungs (~ 10/lung) and snap frozen in liquid nitrogen.

### EZH2 knock down

Two shRNAs targeting distinct regions of EZH2 and a non-targeting sequence (scrambled control) were ligated into the pSilencer™ 4.1 CMV expression vector and transfected into HBEC2. Clones were selected with hygromycin.

### Gene expression

Total RNA was isolated from cell lines and mouse tumors and reverse transcribed using the High Capacity cDNA Reverse Transcription Kit. RT-qPCR was carried out with inventoried TaqMan assays. Experiments were performed in triplicate and normalized to β-Actin or GAPDH using the 2^(−ΔΔ*C*t)^ method.

### Western blot

Protein (50–100 μg) was electrophoretically fractionated, blotted onto a nitrocellulose 0.45 µm membrane, blocked 60 min in 5% non-fat milk, and incubated overnight at 4 °C with primary antibodies specific to H3K9me2, H3K27me3, total histone H3, G9a, EZH2, or β-actin followed by detection with goat anti-rabbit or anti-mouse horseradish peroxidase-conjugated secondary antibody and visualization with SuperSignal® West Pico Chemiluminescent Substrate. Quantitative analysis of band intensity was performed using ImageJ.

### Expression arrays and RNA-seq

Total RNA was converted to cRNA and hybridized using the Illumina Whole-Genome Human HT-12 v4.0 Gene Expression BeadChip. Raw expression data were processed using the *lumi* package in R (v 3.3.2) for variance stabilizing transformation and robust spline normalization and log2 fold changes were calculated to identify differentially expressed genes.

RNA integrity for sequencing was assessed with an Agilent 2100 Bioanalyzer and an RNA Integrity Number extracted from the electropherogram to determine quality. cDNA libraries of 250–300 bp were prepared using the TrueSeq Stranded mRNA Sample Preparation Kit (Illumina). Samples were sequenced at 150 bp paired-end runs at a depth of 20 million reads per sample using an Illumina HiSeq3000.

### Methylation arrays

Bisulfite modified DNA (1 µg) isolated from transformed clones and controls were hybridized to the Infinium HumanMethylation450K Beadchip for methylation arrays. Idat files were exported from Genome Studio (Illumina) and preprocessed using the normal-exponential out-of-band method for background correction with dye-bias normalization from the minfi package in R (3.3.2) to generate *β*-values for 485,577 probes.

### DNA methylation analysis

DNA extraction, modification, and promoter CpG island methylation was studied in transformed HBECs and lung cancer cell lines using COBRA as described [53]. Bisulfite sequencing was used to determine methylation density of promoter regions in methylated genes from HBEC2T as described [11]

### Identification of methylated genes with reduced expression from the TCGA dataset

We analyzed the TCGA dataset generated with the Illumina HM450K Beadchip that interrogated 811 adenocarcinomas and/or squamous cell carcinomas for genome-wide methylation changes. The approach described previously identified 3759 methylated genes with loss of expression ≥ 2-fold for comparison to methylated genes in transformed HBECs [54].

### Chromatin immunoprecipitation

Methylation of histone 3 lysine residues were examined using ChIP as described [11]. ChIP-on-chip H3K27me3 and H3K9me2 was performed using amplified DNA generated from individual ChIP samples by PCR according to Affymetrix’s protocol. Sample fragmentation, labeling, hybridization, and data extraction were performed by the DNA Array Core facility at Johns Hopkins. GeneChip Human Tiling Promoter array (Affymetrix) was used for hybridizations. Analysis was performed using the model-based analysis of tiling array algorithm. Gene promoter-specific ChIP enrichment for the chromatin marks was compared to expression determined by Agilent 44K array.

### Statistical analysis

Our analytic strategy for the HM450K arrays focused on the methylation status of 84,735 CpG oligonucleotide probes within 200 base pairs 5′ of the TSS and extending through exon 1. Methylation analysis was restricted to probes in vehicle treated cell lines with *β*-value < 0.2. Thus, genes methylated in normal HBECs or tissue are not included in our discovery of methylated genes associated with transformation or malignancy. Probes with *β*-values ≥ 0.45 in transformed HBECs were considered to be methylated. For the cancer cell lines, genes with average *β* values ≥ 0.45 across the promoter region were defined as methylated.

Normalized expression microarray data were used to identify gene expression changes ≥ 2-fold in transformed HBECs relative to vehicle treated controls. Genes with ≥ 1.25-fold reduced expression were selected to examine the relationship between reduced expression and promoter hypermethylation in HBECs.

RNA sequencing data from mouse tumors were analyzed using Illumina’s cloud-based genomics-computing environment. Qiagen Ingenuity Pathway Analysis software was used to identify pathways and networks statistically over-represented in the lists of differentially expressed genes from the expression arrays and RNA-seq. Statistical analyses were conducted in SAS 9.4 and R 3.3.2.

## Supplementary Information

**Additional file 1:** Supplemental Methods, Tables, and Figures.
