# Supplementary material for: Chromatin remodeling by the histone methyltransferase EZH2 drives lung pre-malignancy and is a target for cancer prevention
Source: Clin Epigenetics. 2021 Feb 25;13:44. doi: 10.1186/s13148-021-01034-4 (PMC7908796; doi:10.1186/s13148-021-01034-4)
Supplement: Supplementary file 1 — Additional file 1: Supplemental Methods, Tables, and Figures. [file 13148_2021_1034_MOESM1_ESM.docx]

**Chromatin Remodeling Drives Pre-malignancy and is a Target for Prevention**

**Supplemental Materials**

**Methods**

**Cell culture, tumor specimens, and carcinogen exposure**

HBEC1, HBEC2, HBEC13, and HBEC14 immortalized with *hTERT* and *CDK4* were obtained from Drs. Shay and Minna, Southwestern Medical Center, Dallas, TX. Cell culture conditions have been described (10). Tumor-derived cell lines (A549, Calu6, Calu3, H23, H358, H1299, H1435, H1568, H1975, H1993, H2023, H2085, H2170, H2228, HCC827, PC9, SKLU1, SKMES1, and SW900) were obtained from the American Type Culture Collection. Cell line authentication was performed using FTA Sample Collection Kit for Human Cell Authentication (*ATCC*). Cells were tested negative for mycoplasma using MycoProbe Detection Kit (*R&D Systems*).

Benzo(a)pyrene-diolepoxide1 (BPDE) and methylnitrosourea (MNU) were obtained from Drs. Desai and Amin (Penn State) and stored at -20°C in a dry powder form and resuspended in DMSO (Sigma) prior to treatment. HBEC13 and HBEC14 were exposed to 0.05 µM BPDE and 0.5 mM MNU for 1 hr, once a week for 12 weeks, and propagated in keratinocyte specific medium (KSM) containing 15% FBS as described (10).

**Soft agar colony formation**

Soft agar assays were conducted as described previously (10). Cultures were photographed and the colonies with diameters larger than 100 µm were counted using Image software.

**Animal treatment and histopathology**

All animal procedures were conducted under protocols approved by the Institutional Animal Care and Use Committee at Lovelace Respiratory Research Institute, which is accredited by the Association for Assessments and Accreditation of Laboratory Animal Care International. Female A/J mice (n = 100) obtained from Jackson Laboratory (4–6 weeks old) were treated 3 times (every other day, 50 mg/kg, i.p.) with NNK (Chemsyn Science Laboratories) dissolved in saline or with saline alone (0.1 ml). Animals were held for 20 weeks following carcinogen exposure to allow for the development of pre-invasive lesions: alveolar hyperplasias and adenomas (26).  Mice were then separated into 5 groups of 20 mice and treated for 20 weeks with individual or a combination of the agents.  The agents used were DZNep (2.0 mg/kg, i.p. [Cayman]), EPZ6438 (75 mg/kg, i.p. [Cayman]), UNC0642 (5.0 mg/kg, i.p. [Cayman]), a diet rich with 4% omega-3 fish oil (EPA/DHA ethyl esters 45/37 provided by SOLUTEX GC, S.L.), or a control diet calorically balanced with 5% corn oil and 500 IU/g vitamin E. Both diets contained identical kilocalories with respect to percentage of protein, carbohydrate, and fat (Supplemental Table 6). Diets were prepared by Envigo Teklad (New Jersey), vacuum packed in aliquots sufficient for one week, stored at -20°C prior to use, and then moved to 4°C for each weeks’ allotment. Mice were treated three times each week (Monday, Wednesday, Friday) with the combination of EPZ6438/UNC0642 to avoid toxicity by UNC0642 or five times each week with DZNep or vehicle (DMSO). Mice were fed control or 4% omega 3 fish oil diet continuously with fresh diet provided every 2 days.

Mice (12–15/group) were sacrificed by exsanguination; lungs were inflated and fixed with 4% buffered paraformaldehyde for 18–24 h and then transferred to 70% ethanol for routine histological processing and staining of paraffin sections with hematoxylin and eosin. A single standardized section was prepared from all lungs that included the five lung lobes. Pulmonary lesions were classified as hyperplasia or neoplasia (adenoma or carcinoma) as described (25, 26). Following sacrifice of the remaining 5 mice/group, tumors were removed from lungs (~10/lung) and snap frozen in liquid nitrogen.

**EZH2 knock down**

Two siRNAs targeting distinct regions of EZH2 (ID#2140221 and146511 [Ambion]) and a non-targeting sequence (scrambled siRNA ID# negative control No. 1) were ligated into the pSilencer^TM^ 4.1 CMV expression vector and transfected into HBEC2. Clones were selected with hygromycin.

**Gene expression**

Total RNA was isolated from cell lines and mouse tumors and quantified using a NanoDrop 2000 spectrophotometer and Qubit 2.0 Fluorometer (Life Technologies). RNA (1 μg) was reverse transcribed using the High Capacity cDNA Reverse Transcription Kit (Life Technologies). RT-qPCR was carried out with inventoried TaqMan assays (Life Technologies). Experiments were performed in triplicate and normalized to β-Actin or GAPDH using the 2^(−ΔΔ^*^C^*^t)^ method.

**Western blot**

Cells or tissue samples were lysed using RIPA buffer and 50-100 μg of protein was electrophoretically fractionated on 4–15% Mini-PROTEAN® TGX™ Precast 10 wells gel (Bio-Rad), blotted onto a nitrocellulose 0.45 µm membrane (Bio-Rad), blocked 60 min in 5% non-fat milk, and incubated overnight at 4°C with primary antibodies specific to H3K9me2 (*Abcam*), H3K27me3 (Abcam), total histone H3 (Abcam), G9a (Becton Dickinson), EZH2 (Cell Signaling), or β-actin (Cell Signaling) followed by detection with goat anti-rabbit or anti-mouse horseradish peroxidase-conjugated secondary antibody (Cell Signaling) and visualization with SuperSignal® West Pico Chemiluminescent Substrate. Quantitative analysis of band intensity was performed using ImageJ.

**Expression arrays and RNA-seq**

Total RNA was converted to cRNA and hybridized using the Illumina Whole-Genome Human HT-12 v4.0 Gene Expression BeadChip. Raw expression data were processed using the *lumi* package in R (v 3.3.2) for variance stabilizing transformation and robust spline normalization and log2 fold changes were calculated to identify differentially expressed genes.

RNA integrity for sequencing was assessed with an Agilent 2100 Bioanalyzer and a RNA Integrity Number extracted from the electropherogram to determine quality. cDNA libraries of 250–300 bp were prepared using the TrueSeq Stranded mRNA Sample Preparation Kit (Illumina). Samples were sequenced at 150 bp paired-end runs at a depth of 20 million reads per sample using an Illumina HiSeq3000 (Novogene).

**Methylation arrays**

Bisulfite modified DNA (1µg) isolated from transformed clones and controls were hybridized to the Infinium HumanMethylation450K Beadchip (Illumina) for methylation arrays. Idat files were exported from Genome Studio (Illumina) and preprocessed using the normal-exponential out-of-band method for background correction with dye-bias normalization from the minfi package in R (3.3.2) to generate β-values for 485,577 probes.

**DNA methylation analysis**

DNA extraction, modification, and promoter CpG island methylation was studied in transformed HBECs and lung cancer cell lines using Combined Bisulfite Modification and Restriction Analysis (COBRA) as described (53). Bisulfite sequencing was used to determine methylation density of promoter regions in methylated genes from transformed HBEC2 as described (11).

**Identification of methylated genes with reduced expression from the TCGA dataset**

We analyzed the TCGA dataset generated with the Illumina HM450K Beadchip array that interrogated 811 adenocarcinomas and/or squamous cell carcinomas for genome-wide methylation changes. The approach described previously identified 3,759 methylated genes with loss of expression ≥2-fold for comparison to methylated genes in transformed HBECs (54).

**Chromatin immunoprecipation.**

Methylation of histone 3 lysine residues were examined using ChIP as described (11). ChIP grade antibodies specific for total Histone H3, H3K9me2 (Abcam), and H3K27me3 (Cell Signaling) were used to capture protein–DNA complexes. Rabbit and mouse IgGs were used for isotype controls. Results were generated by qPCR performed in triplicate using Power SYBR® Green PCR Master Mix (Applied Biosystems). Primer sequences are available upon request. Results were quantified using a 2^(-ΔΔCt)^ method.

ChIP-on-chip H3K27me3 and H3K9me2 was performed using amplified DNA generated from individual ChIP samples by PCR according to Affymetrix’s protocol. Sample fragmentation, labeling, hybridization, and data extraction were performed by the DNA Array Core facility at Johns Hopkins. GeneChip Human Tiling Promoter array (Affymetrix) was used for hybridizations. Analysis was performed using the model-based analysis of tiling array algorithm. Gene promoter-specific ChIP enrichment for the chromatin marks was compared to expression determined by Agilent 44K array.

**Statistical analysis.**

Our analytic strategy for the HM450K arrays focused on the methylation status of 84,735 CpG oligonucleotide probes within 200 base pairs 5' of the TSS and extending through exon 1. Methylation analysis was restricted to probes in vehicle treated cell lines with β-value <0.2. Probes with β-values ≥ 0.45 in transformed HBECs were considered to be methylated. For the cancer cell lines, genes with average β values ≥ 0.45 across the promoter region were defined as methylated.

Normalized expression microarray data were used to identify gene expression changes ≥2-fold in transformed HBECs relative to vehicle treated controls. Genes with ≥1.25-fold reduced expression were selected to examine the relationship between reduced expression and promoter hypermethylation in HBECs.

RNA sequencing data from the mouse tumors were analyzed using Illumina’s cloud-based genomics-computing environment. Sequencing reads were filtered and trimmed using Bowtie, aligned to a mouse reference genome (GRCm38) using STAR, and quantified using Salmon through Illumina’s RNA-Seq Alignment app v2.0. The count-based statistical method DESeq2 in Illumina’s RNA-Seq Differential Expression app v1.01 was used to identify differences (fold changes) between treated tumors relative to controls. DeSeq2 uses the Wald test for significance testing and Benjamini-Hochberg method to determine the false discovery rate (FDR).

Qiagen Ingenuity Pathway Analysis software was used to identify pathways and networks statistically over-represented in the lists of differentially expressed genes from the expression arrays and RNA-seq. Statistical analyses were conducted in SAS 9.4 and R 3.3.2

**Supplemental Table 1. HBECT methylated genes also methylated in cell lines and/or TCGA tumors**

| **Gene_name** | **Cancer cell lines** | **TCGA tumors** | **Gene_name** | **Cancer cell lines** | **TCGA tumors** |
| --- | --- | --- | --- | --- | --- |
| ALDH3A1 | * | * | LEPREL1 | * | * |
| ARHGDIB | * | * | MGAT1 | * | * |
| ARHGEF10 | * | * | MYLK | * | * |
| AUTS2 | * | * | NCOR2 | * | * |
| CC2D2A | * | * | NDRG4 | * | * |
| CDC42EP5 | * | * | NUAK1 | * | * |
| CHRNB1 | * | * | PAK6 | * | * |
| COL16A1 | * | * | PCSK9 | * | * |
| CSPG4 | * | * | PLTP | * | * |
| CYGB | * | * | PPP1R13L | * | * |
| D4S234E | * | * | PSTPIP2 | * | * |
| DKK3 | * | * | RAI1 | * | * |
| FAM110A | * | * | ROBO3 | * | * |
| FAM131A | * | * | SDR42E1 | * | * |
| FAM176A | * | * | SEMA3F | * | * |
| FCGRT | * | * | SGK1 | * | * |
| FLI1 | * | * | SLC27A3 | * | * |
| FRMD6 | * | * | SLIT3 | * | * |
| GAL3ST4 | * | * | SOX15 | * | * |
| GRB7 | * | * | SPOCK1 | * | * |
| GSTM1 | * | * | SSTR2 | * | * |
| HAS3 | * | * | TACSTD2 | * | * |
| HS3ST1 | * | * | TMEM40 | * | * |
| IGFBP2 | * | * | TNFRSF6B | * | * |
| IRF6 | * | * | TSPAN9 | * | * |
| KIAA0247 | * | * | ZNF529 | * | * |
| KIAA1217 | * | * | ZNF532 | * | * |
| KRT19 | * | * |  |  |  |

*Indicates methylated in cell lines or TCGA tumors.

**Supplemental Table 1 continued**

| **Gene_name** | **Cancer cell lines** | **TCGA tumors** | **Gene_name** | **Cancer cell lines** | **TCGA tumors** |
| --- | --- | --- | --- | --- | --- |
| AFAP1L2 | * |  | KIAA1671 | * |  |
| AGR2 | * |  | KRT15 | * |  |
| ANKRD22 | * |  | KRT23 | * |  |
| ARHGEF16 | * |  | KRT5 | * |  |
| ARTN | * |  | KRT6A | * |  |
| BCL6 | * |  | KRT6B | * |  |
| CBLC | * |  | KRTCAP3 | * |  |
| CCDC92 | * |  | LCOR | * |  |
| CELSR3 | * |  | LMTK3 | * |  |
| CLDN23 | * |  | NCK2 | * |  |
| CLDN7 | * |  | NDST1 | * |  |
| CLSTN1 | * |  | PADI3 | * |  |
| COL17A1 | * |  | PAK1 | * |  |
| CTSK | * |  | PAQR7 | * |  |
| CYFIP1 | * |  | PER2 | * |  |
| DQX1 | * |  | PLEKHA4 | * |  |
| DSG3 | * |  | PRICKLE2 | * |  |
| EHF | * |  | PROM2 | * |  |
| ELMO3 | * |  | PRSS8 | * |  |
| EPN3 | * |  | PTK6 | * |  |
| EVPL | * |  | RAB25 | * |  |
| EXTL3 | * |  | RAB7A | * |  |
| FAR2 | * |  | RNASE7 | * |  |
| FGFBP1 | * |  | RTKN | * |  |
| GALNT6 | * |  | S100A14 | * |  |
| GBP6 | * |  | SEMA4A | * |  |
| GDF15 | * |  | SH3BGRL3 | * |  |
| GJB5 | * |  | SLC20A2 | * |  |
| GNA15 | * |  | SPDEF | * |  |
| GPR110 | * |  | STAP2 | * |  |
| GPR56 | * |  | SULF1 | * |  |
| GPX2 | * |  | SVIL | * |  |
| GRHL2 | * |  | SYTL1 | * |  |
| GSN | * |  | TAGLN | * |  |
| HMBOX1 | * |  | TANC1 | * |  |
| IL1B | * |  | TMEM30B | * |  |
| INPP4B | * |  | VAMP8 | * |  |
| ITGB4 | * |  | ZNF605 | * |  |
| ITGB6 | * |  |  |  |  |

**Supplemental Table 1 continued**

| **Gene_name** | **Cancer cell lines** | **TCGA tumors** | **Gene_name** | **Cancer cell lines** | **TCGA tumors** |
| --- | --- | --- | --- | --- | --- |
| ABCB6 |  | * | LARP6 |  | * |
| ANGPTL4 |  | * | MAD2L2 |  | * |
| ANXA3 |  | * | MARVELD2 |  | * |
| ARPC1B |  | * | POPDC3 |  | * |
| CTSL2 |  | * | SERTAD2 |  | * |
| FAM64A |  | * | TP53INP1 |  | * |
| H1F0 |  | * | TPM1 |  | * |
| HBP1 |  | * | ZNF470 |  | * |
| JUP |  | * | ZSCAN21 |  | * |

**Supplemental Table 2. HBECT unmethylated genes with reduced expression that become methylated in cancer cell lines and/or TCGA tumors**

| **Gene_name** | **Cancer cell lines** | **TCGA tumors** | **Gene_name** | **Cancer cell lines** | **TCGA tumors** |
| --- | --- | --- | --- | --- | --- |
| ABLIM1 | * | * | CKMT1B | * |  |
| ACTA2 | * |  | CLIP4 | * | * |
| ADAMTS1 | * | * | CNTNAP2 | * | * |
| ADM |  | * | COL7A1 | * | * |
| ADRB2 |  | * | CPE |  | * |
| AKR1B10 | * |  | CSF2 | * |  |
| ALDH1A3 |  | * | CSTA | * |  |
| ALDH7A1 |  | * | CTDSPL |  | * |
| ALDOC | * | * | CTNNBIP1 | * |  |
| ANKRD33 | * |  | CUX2 | * |  |
| ANKRD57 | * |  | CYBRD1 | * | * |
| ANXA1 | * |  | CYP1B1 |  | * |
| ANXA8L2 | * |  | CYYR1 | * | * |
| ARHGAP22 |  | * | DFNA5 | * | * |
| ARHGEF3 | * |  | DLL1 |  | * |
| ARHGEF5 | * |  | DSC3 | * | * |
| ARHGEF5L | * |  | DST |  | * |
| ARID5B |  | * | DUSP22 | * |  |
| ARRDC4 |  | * | ELF1 | * |  |
| BLCAP | * |  | FADS1 |  | * |
| C10orf116 | * | * | FAM113B | * |  |
| C10orf35 |  | * | FAM84B |  | * |
| C16orf74 | * | * | FAP | * |  |
| C1orf116 | * |  | FAT2 | * |  |
| C21orf7 | * |  | FBN2 | * | * |
| C6orf105 | * |  | FCRLA | * |  |
| C6orf115 |  | * | FEZ1 | * | * |
| CA12 | * | * | FKBP11 | * | * |
| CALD1 | * |  | FLRT2 | * | * |
| CCND2 | * | * | FOLR3 | * |  |
| CD70 | * |  | FOXA2 | * | * |
| CDH1 |  | * | FOXQ1 |  | * |
| CDH13 | * | * | FSCN1 |  | * |
| CDH3 | * |  | FST | * | * |
| CDK6 |  | * | GALNT5 | * | * |
| CELSR2 | * |  | GJA1 | * | * |
| CKMT1A | * |  | GPR68 | * |  |

*Indicates methylated in cell lines or TCGA tumors.

**Supplemental Table 2 continued**

| **Gene_name** | **Cancer cell lines** | **TCGA tumors** | **Gene_name** | **Cancer cell lines** | **TCGA tumors** |
| --- | --- | --- | --- | --- | --- |
| GPR87 | * |  | LPXN | * |  |
| GPSM2 |  | * | LRRC8A | * |  |
| GPX3 | * | * | LTBP2 | * | * |
| GRAMD3 | * |  | MAL2 |  | * |
| GSTP1 |  | * | MAPK13 | * |  |
| H2AFY2 | * | * | MATN2 |  | * |
| HCLS1 | * |  | MIR205 | * |  |
| HES2 |  | * | MLLT11 | * | * |
| HSD17B8 | * | * | MMP1 | * |  |
| IFI27 | * |  | MN1 | * | * |
| IGFBP3 |  | * | MT1A | * | * |
| IGFBP7 | * |  | MTSS1 |  | * |
| IL1A | * |  | MX1 | * | * |
| IL20RB | * |  | NAPRT1 | * | * |
| INPP5D | * |  | NDRG1 | * |  |
| IRX4 | * |  | NET1 | * | * |
| JAKMIP2 | * | * | NFE2L2 |  | * |
| JAM3 | * | * | NHLH2 | * | * |
| KANK4 | * | * | NRBP2 | * |  |
| KCNK1 |  | * | NT5E |  | * |
| KIAA1522 | * |  | NUPR1 | * |  |
| KRT14 | * |  | PAFAH1B3 |  | * |
| KRT16 | * |  | PALLD | * | * |
| KRT17 | * |  | PCSK9 | * | * |
| KRT6C | * |  | PDPN | * | * |
| KRT7 | * | * | PIK3IP1 |  | * |
| LAMA3 | * |  | PKP1 | * | * |
| LAMB3 | * |  | PLCH2 | * |  |
| LCN2 | * |  | PPP1R3C |  | * |
| LIMA1 |  | * | PRKCDBP | * | * |
| LIPG | * |  | PROCR |  | * |
| LITAF | * |  | PRRG2 | * |  |
| LOC100129550 | * |  | PRSS23 |  | * |
| LOC728643 | * |  | PTGER4 |  | * |
| LOX | * | * | PTPRM |  | * |
| LPAR5 | * |  | PYCARD | * | * |
| LPHN2 |  | * | RAB32 |  | * |

**Supplemental Table 2 continued**

| **Gene_name** | **Cancer cell lines** | **TCGA tumors** | **Gene_name** | **Cancer cell lines** | **TCGA tumors** |
| --- | --- | --- | --- | --- | --- |
| RAB38 | * | * | STAT4 | * |  |
| RHOD | * | * | SULF2 | * | * |
| RICS | * |  | THBS2 | * | * |
| RNF145 |  | * | TICAM2 | * | * |
| S100A16 | * |  | TINAGL1 | * | * |
| S100A2 | * |  | TMC6 | * |  |
| S100P | * |  | TMED10P | * |  |
| SAMD9 | * |  | TMEM154 | * | * |
| SCG5 | * |  | TMEM158 |  | * |
| SDK2 | * | * | TMSL3 | * |  |
| SEMA3C |  | * | TNC | * |  |
| SERPINB13 | * |  | TNFSF10 | * |  |
| SERPINB2 | * |  | TNS3 | * | * |
| SERPINB5 | * |  | TP63 | * |  |
| SERPINB7 | * |  | TPD52L1 |  | * |
| SERPINE1 | * | * | TRIM22 | * |  |
| SERPINE2 | * | * | TXNIP |  | * |
| SFRP1 | * | * | UBE2E2 |  | * |
| SH2D3A | * |  | UCN2 | * |  |
| SH3D19 | * |  | VEGFC | * |  |
| SIRPA |  | * | VIM |  | * |
| SLC2A3 | * | * | VSNL1 | * | * |
| SLC47A2 | * |  | WARS | * | * |
| SLC7A5 |  | * | WDR66 | * |  |
| SLC9A1 | * |  | XDH | * |  |
| SNAI2 |  | * | YPEL5 |  | * |
| SNCA | * | * | ZBED2 | * |  |
| SOCS2 |  | * | ZMIZ1 | * | * |
| SOX7 | * | * | ZNF219 |  | * |
| SPARC | * | * | ZNF395 |  | * |
| SPATS2L | * | * | ZNF738 | * |  |
| SPOCD1 | * |  | ZNF83 |  | * |
| SQLE |  | * | ZSCAN18 | * | * |
| ST14 | * |  |  |  |  |

**Supplemental Table 3. HBEC2T genes enriched for H3K27me3**

| **Gene** |  | **Gene** |  | **Gene** |  |
| --- | --- | --- | --- | --- | --- |
| DHCR7 |  | DQX1 |  | C12orf5 |  |
| MAPKAPK3 |  | ELMO3 |  | C1orf190 |  |
| MST1R |  | EPB41L4A |  | C3orf14 |  |
| SNAI2 |  | EPHA1 |  | C9orf95 |  |
| PRDX5 |  | GJB3 |  | CA2 |  |
| SURF2 |  | HBEGF |  | CALM1 |  |
| TOMM40L |  | IL27RA |  | CBR4 |  |
| ABCB6 |  | KIAA0284 |  | CCNG2 |  |
| CHML |  | KIAA0649 |  | CD1D |  |
| KIAA0753 |  | KLRC4 |  | CDC42BPA |  |
| RAB25 |  | KRT14 |  | CDH1 |  |
| C16orf74 |  | LTB |  | CHMP4C |  |
| RNASE7 |  | MDFI |  | CKMT1A |  |
| PLXNB1 |  | MGAT4A |  | CLDN7 |  |
| SLC38A5 |  | MOBKL2B |  | COL16A1 |  |
| FYB |  | MYCBPAP |  | CRIPT |  |
| MCC |  | OCIAD2 |  | CYFIP1 |  |
| SLC37A1 |  | PKP1 |  | DUOX1 |  |
| SOCS6 |  | POLI |  | DUSP5 |  |
| USP33 |  | PWP2 |  | ENTPD3 |  |
| ACLY |  | RNF128 |  | EPHB4 |  |
| BTBD11 |  | SERPINF1 |  | EPSTI1 |  |
| C9orf5 |  | SESN3 |  | EXPH5 |  |
| CTSH |  | SH3YL1 |  | EZH2 |  |
| FEZ1 |  | STYK1 |  | FAM117A |  |
| ITGB6 |  | SYK |  | FAM83A |  |
| LPXN |  | TMPRSS4 |  | FASN |  |
| MICALL1 |  | ABCG1 |  | FBXO2 |  |
| NLN |  | ALDH7A1 |  | FBXO5 |  |
| NUP62CL |  | AMOTL2 |  | FLRT2 |  |
| PELI1 |  | ANKRD27 |  | FNBP1L |  |
| AKR1C1 |  | ANKRD28 |  | FXYD3 |  |
| CDKL3 |  | APP |  | GJB5 |  |
| CEBPG |  | ARPC1B |  | GLUL |  |
| CELSR1 |  | ASPM |  | GPD2 |  |
| CTNNBIP1 |  | BACE1 |  | GPR56 |  |
| CYP27B1 |  | BLCAP |  | GPX2 |  |
| DAB2IP |  | BNC1 |  | GRK5 |  |

**Supplemental Table 3 continued**

| **Gene** |  | **Gene** |  | **Gene** |  |
| --- | --- | --- | --- | --- | --- |
| GSTO2 |  | PTPN3 |  | ATP7A |  |
| HBP1 |  | PVRL1 |  | BEX2 |  |
| HSPA4L |  | ROS1 |  | BTN3A2 |  |
| IFI44L |  | RPS6KA1 |  | C14orf149 |  |
| IFIH1 |  | RUNX2 |  | C16orf70 |  |
| IGSF9 |  | SCD |  | C6orf115 |  |
| KCNJ15 |  | SERPINB8 |  | C6orf192 |  |
| KIF18A |  | SH2D3A |  | C6orf48 |  |
| KLRC3 |  | SHROOM2 |  | C8orf48 |  |
| LIMA1 |  | SLC37A2 |  | CA14 |  |
| LIPG |  | SNX1 |  | CAMP |  |
| LOXL3 |  | SOCS2 |  | CD274 |  |
| LPHN2 |  | SPHK1 |  | CD83 |  |
| LRRC8C |  | SRCRB4D |  | CD99L2 |  |
| LSR |  | SYT1 |  | CEP76 |  |
| MALL |  | TMC6 |  | CLEC2B |  |
| MAP2K1 |  | TMEM54 |  | CORO2A |  |
| MAPK13 |  | TMTC2 |  | CPA4 |  |
| METTL7A |  | TTC32 |  | DHRS7 |  |
| MIS12 |  | UGCG |  | DHRS9 |  |
| MSRB2 |  | UST |  | DIXDC1 |  |
| MTL5 |  | VDR |  | DLC1 |  |
| NBL1 |  | VSNL1 |  | DNAJC7 |  |
| NCOA1 |  | ZNF462 |  | DSTN |  |
| NDFIP2 |  | ZSCAN18 |  | EFEMP1 |  |
| NDRG1 |  | ABI3BP |  | EHD4 |  |
| NDUFB2 |  | ADRB2 |  | ELF2 |  |
| NHSL1 |  | AIFM2 |  | ERF |  |
| NPDC1 |  | AMPD3 |  | EVI2A |  |
| OSBPL10 |  | ANKRD50 |  | EYA1 |  |
| PAK6 |  | ANP32C |  | FAM110C |  |
| PBXIP1 |  | ANXA10 |  | FAM57A |  |
| PHLDA1 |  | AP1M2 |  | FAM83H |  |
| PLAU |  | APLP2 |  | FAP |  |
| PPP1R3C |  | ARID3B |  | FBXW7 |  |
| PQLC2 |  | ARL3 |  | FEM1B |  |
| PTGFRN |  | ASMTL |  | FGF1 |  |
| PTGS2 |  | ATP6V1G1 |  | FRMD4B |  |

**Supplemental Table 3 continued**

| **Gene** |  | **Gene** |  | **Gene** |  |
| --- | --- | --- | --- | --- | --- |
| FZD8 |  | NEK2 |  | SEC31B |  |
| GK |  | NPL |  | SEPW1 |  |
| GLOD4 |  | ORAOV1 |  | SERPINE2 |  |
| GPR81 |  | ORMDL1 |  | SESN1 |  |
| GRAMD1C |  | PBX2 |  | SETBP1 |  |
| H1F0 |  | PCDH7 |  | SH3BGRL |  |
| HISPPD2A |  | PCNX |  | SHPRH |  |
| HLA-G |  | PDIA3 |  | SLAMF7 |  |
| HOMER2 |  | PEG3 |  | SLC16A3 |  |
| HOOK1 |  | PFTK1 |  | SLC1A3 |  |
| HS2ST1 |  | PGRMC2 |  | SMO |  |
| HSD17B11 |  | PHEX |  | SNCA |  |
| HSD17B7P2 |  | PHF21A |  | SNX10 |  |
| HTATIP2 |  | PHGDH |  | SPRR1B |  |
| IL1RAP |  | PHYH |  | TAGLN3 |  |
| IL4R |  | PLEKHN1 |  | THSD1 |  |
| IMPA2 |  | PNKD |  | TIGD7 |  |
| IRX4 |  | POU2F2 |  | TIPARP |  |
| JUP |  | PPARGC1B |  | TMCC3 |  |
| LEPR |  | PRDM2 |  | TMEM102 |  |
| LEPRE1 |  | PRKCE |  | TMEM14B |  |
| LETM2 |  | PTPLA |  | TNFRSF10A |  |
| MAB21L1 |  | PTPN13 |  | TOMM34 |  |
| MARVELD1 |  | PTPRF |  | TREM2 |  |
| MARVELD2 |  | QPCT |  | TRIM4 |  |
| MBNL2 |  | RB1CC1 |  | TSPAN13 |  |
| MESDC1 |  | RBBP8 |  | UPF3B |  |
| MKI67 |  | RDH11 |  | USP43 |  |
| MMP9 |  | RGS2 |  | VEGFC |  |
| MND1 |  | RHOBTB2 |  | WNT7A |  |
| MPDZ |  | RLN1 |  | ZBTB20 |  |
| MRPL33 |  | RNF145 |  | ZFP14 |  |
| MYO10 |  | S100A11 |  | ZNF608 |  |

**Supplemental Table 4. HBEC2T genes enriched for H3K9me2**

| **Gene** |  | **Gene** |  | **Gene** |  |
| --- | --- | --- | --- | --- | --- |
| C8orf74 |  | NIPAL2 |  | COBRA1 |  |
| FUT4 |  | PGPEP1 |  | CYP2E1 |  |
| TRMU |  | REPIN1 |  | GFER |  |
| PANX2 |  | TSSK6 |  | GPR78 |  |
| A1BG |  | ZIC4 |  | GRAMD1A |  |
| AGBL4 |  | ZIC5 |  | NEFM |  |
| C9orf172 |  | C20orf177 |  | NLRP14 |  |
| MESDC2 |  | C6orf145 |  | NR2F1 |  |
| NECAB3 |  | CCL11 |  | POM121L12 |  |
| ADAMTS20 |  | COX5A |  | RBP1 |  |
| EXOC2 |  | EHBP1 |  | RNF123 |  |
| FAM100B |  | FOXRED2 |  | RPRM |  |
| KCNG3 |  | KIAA1383 |  | SLC26A6 |  |
| MIR759 |  | MTL5 |  | SSTR5 |  |
| PPP3CB |  | PRLHR |  | TGIF1 |  |
| SHE |  | PSD4 |  | ZNF497 |  |
| BCCIP |  | RDH13 |  | ANKRD30A |  |
| BOLL |  | RNU1-8 |  | ARSK |  |
| SDR16C6 |  | RNU7-19P |  | BMI1 |  |
| ASPDH |  | SIM2 |  | CUX1 |  |
| C9orf163 |  | SLC47A1 |  | FAM173A |  |
| FOXL2 |  | WDR8 |  | FARP1 |  |
| NUBP2 |  | C14orf101 |  | GPR6 |  |
| RECK |  | DMRT1 |  | KBTBD5 |  |
| SLC12A5 |  | FSD1 |  | KLF17 |  |
| TMEM133 |  | GNASAS |  | MAPK15 |  |
| TOP3B |  | GZF1 |  | NAT2 |  |
| DNAH9 |  | HSPA7 |  | NPHS2 |  |
| GMEB2 |  | KCNA2 |  | ONECUT2 |  |
| KCNV2 |  | PRDM16 |  | OPA3 |  |
| MFHAS1 |  | SCARNA8 |  | POU4F2 |  |
| STIM2 |  | SPACA1 |  | RAX2 |  |
| C16orf70 |  | ZNF358 |  | RFTN1 |  |
| CHAT |  | ARHGDIG |  | THNSL1 |  |
| COPE |  | C17orf82 |  | TRIML2 |  |
| DCHS2 |  | C5orf38 |  | ACTL7B |  |
| FBXW5 |  | CCDC36 |  | BIRC7 |  |
| LEMD2 |  | CHST2 |  | C16orf13 |  |

**Supplemental Table 4 continued**

| **Gene** |  | **Gene** |  | **Gene** |  |
| --- | --- | --- | --- | --- | --- |
| C1orf150 |  | PRR18 |  | ZNF135 |  |
| C7orf70 |  | SPESP1 |  | ASPHD1 |  |
| C9orf142 |  | VPS28 |  | C14orf39 |  |
| CHGA |  | ZMYM2 |  | C7orf50 |  |
| FGF12 |  | C12orf37 |  | C9orf107 |  |
| ICA1 |  | IRF2BP1 |  | FANCI |  |
| KCNK18 |  | NHLRC1 |  | PROX1 |  |
| MEX3B |  | ROCK1 |  | SLC6A5 |  |
| NEK11 |  | SLC2A4RG |  | VWC2 |  |
| PM1H |  | SPEF1 |  |  |  |

**Supplemental Table 5. PRC2 regulated genes with altered expression in
DZNep treated mouse lung tumors.**

| **Gene** | | | | |
| --- | --- | --- | --- | --- |
| ADM | GPR83 | KCNH5 | PLCG2 | SNCAIP |
| ALDH1A2 | HBA1 | LMO2 | POU3F2 | SNRPE |
| ALK | HES7 | MAB21L2 | PPARG | SOX9 |
| B3GNT5 | HOXA10 | MKKS | RET | STXBP5L |
| BFSP1 | HOXA11 | NCAM1 | RGMA | STXBP6 |
| CDH8 | HOXA5 | NDRG1 | SCRT1 | TAL1 |
| CYP2J2 | ICAM4 | NETO1 | SFMBT2 | TP73 |
| EPHA10 | IL10RA | NXPH2 | SFTPC | UPB1 |
| GATA3 | INHBB | PCSK2 | SIM2 | VSIG2 |
| GBX2 | KCNG1 | PDGFRA | SIX3 | WNK4 |

**Supplemental Table 6. Control and Lovaza Diets**

| **Selected Nutrient Information** | **Control Diet** | **Lovaza Diet** |
| --- | --- | --- |
| Protein, % by weight | 12.4 | 12.4 |
| Carbohydrate, % by weight | 67.3 | 67.3 |
| Fat, % by weight | 5.1 | 5.1 |
| **Ingredient** | **Control Diet (g/kg)** | **Lovaza Diet (g/kg)** |
| Casein | 140.0 | 140.0 |
| L-cystine | 1.8 | 1.8 |
| Corn starch | 475.0 | 475.0 |
| Maltodextrin | 145.0 | 145.0 |
| Sucrose | 90.0 | 90.0 |
| Corn oil | 50.0 | 10.0 |
| Cellulose | 50.0 | 50.0 |
| Mineral mix, AIN-93M-MX | 35.0 | 35.0 |
| Vitamin mix, AIN-93-VX | 10.0 | 10.0 |
| Vitamin E, 500 IU/g | 0.14 | 0.0 |
| Choline Bitartrate | 2.5 | 2.5 |
| TBHQ antioxidant | 0.01 | 0.01 |
| Red food color | 0.1 | 0.0 |
| Green food color | 0.0 | 0.1 |
| SX85EE+, EPA/DHA ethyl esters 45/37 | 0.0 | 40.0 |

**
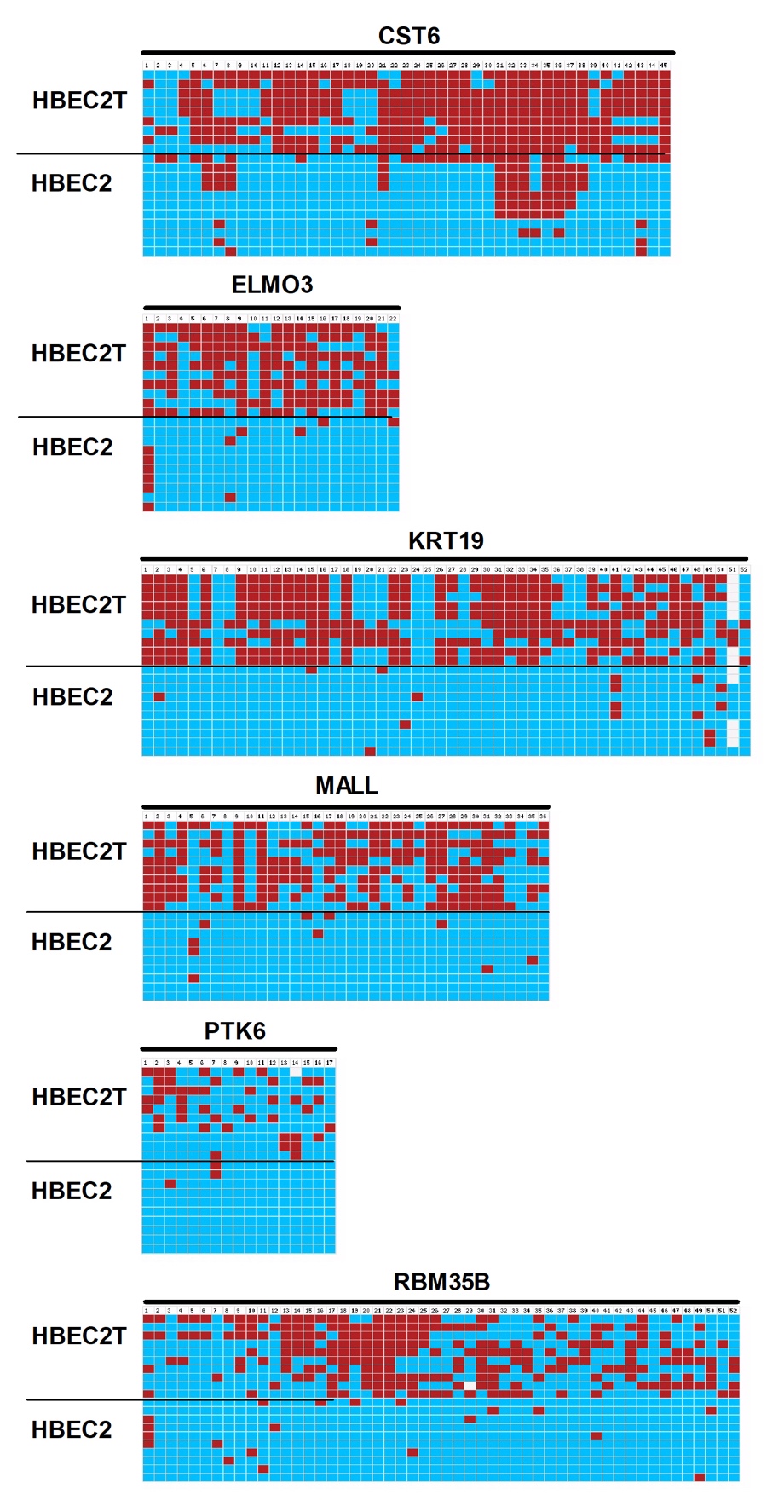
**

**Supplemental Figure 1.** Bisulfite sequencing of CpGs from CST6, ELMO3, KRT19, MALL, PTK6, and RBM35B gene promoter regions from HBEC2T and HBEC2. Methylation state of individual CpGs for 10 clones each are depicted for each gene. Blue, red, and white colored boxes indicate CpGs that are unmethylated, methylated or undetermined.


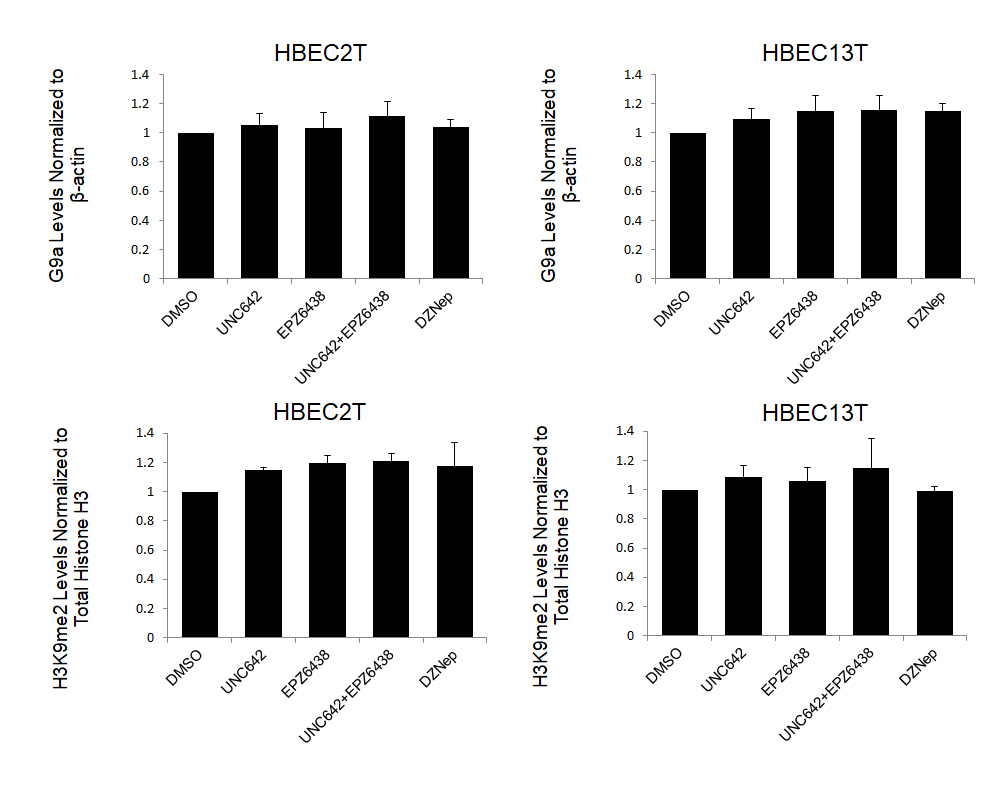


**Supplemental Figure 2.** Densitometry quantitation of G9a and H3K9me2 levels in HBEC2T and HBEC13T cells treated with chromatin remodeling agents.


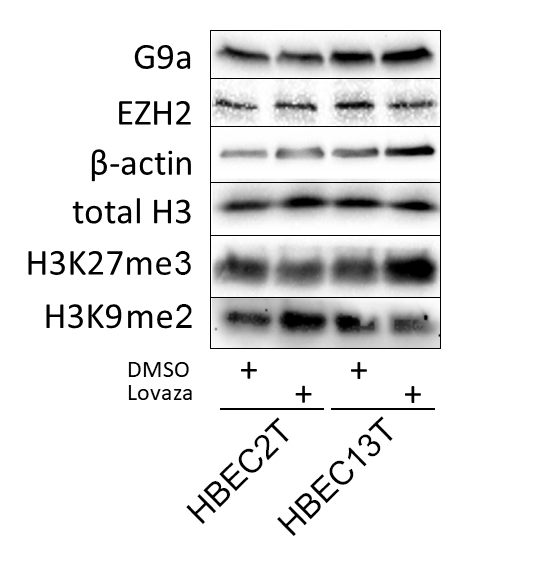


**Supplemental Figure 3.** Western blot showing the effect of treatment of HBEC2T and HBEC13T for 8 days with DMSO or Lovaza on levels of G9a, EZH2, β-actin, total histone H3, H3K27me3, and H3K9me2.


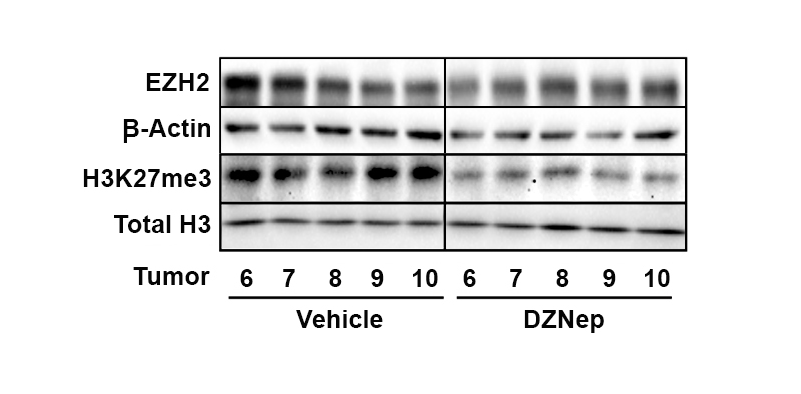


**Supplemental Figure 4.** Western blot showing the levels of EZH2, β-actin, H3K27me3 and total histone H3 in tumors from additional vehicle and DZNep treated mice.
